# The Window Matters: A Systematic Review of Time Restricted Eating Strategies in Relation to Cortisol and Melatonin Secretion

**DOI:** 10.3390/nu13082525

**Published:** 2021-07-23

**Authors:** Shreya Chawla, Spyridon Beretoulis, Aaron Deere, Dina Radenkovic

**Affiliations:** 1 Faculty of Life Sciences and Medicine, King’s College London, London SE1 1UL, UK; shreya.chawla@kcl.ac.uk; 2 HOOKE London owned by Health Longevity Optimisation Ltd., London EC1V 3QJ, UK; spiros@hooke.london (S.B.); aaron@hooke.london (A.D.); 3 Buck Institute for Research on Aging, Novato, CA 94945, USA; 4 Guy’s and St Thomas’ Hospital, Westminster Bridge Road, London SE1 7EH, UK

**Keywords:** time restricted eating, Ramadan, circadian rhythm, chrono-nutrition, intermittent fasting, time restricted feeding, cortisol, melatonin

## Abstract

Time-Restricted Eating is an eating pattern based on the circadian rhythm which limits daily food intake (usually to ≤12 h/day), unique in that no overt restriction is imposed on the quality, nor quantity, of food intake. This paper aimed to examine the effects of two patterns of TRE, traditional TRE, and Ramadan fasting, on two markers of circadian rhythm, cortisol and melatonin. PubMed and Web of Science were searched up to December 2020 for studies examining the effects of time restricted eating on cortisol and melatonin. Fourteen studies met our inclusion criteria. All Ramadan papers found statistically significant decrease in melatonin (*p* < 0.05) during Ramadan. Two out of the three Ramadan papers noted an abolishing of the circadian rhythm of cortisol (*p* < 0.05). The non-Ramadan TRE papers did not examine melatonin, and cortisol changes were mixed. In studies comparing TRE to control diets, Stratton et al. found increased cortisol levels in the non-TRE fasting group (*p* = 0.0018) and McAllister et al. noted no difference. Dinner-skipping resulted in significantly reduced evening cortisol and non-significantly raised morning cortisol. Conversely, breakfast skipping resulted in significantly reduced morning cortisol. This blunting indicates a dysfunctional HPA axis, and may be associated with poor cardio-metabolic outcomes. There is a paucity of research examining the effects of TRE on cortisol and melatonin. The contrasting effect of dinner and breakfast-skipping should be further examined to ascertain whether timing the feeding window indeed has an impact on circadian rhythmicity.

## 1. Introduction

Time-Restricted Eating (TRE) is an eating pattern based on the circadian rhythm that gives the body a daily fasting period. This involves limiting daily food intake to 8–12 h with an intention to prolong the time spent in a fasting state [1]. This form of fasting is unique as no overt restriction is imposed on the quality, nor quantity, of food intake [2]. TRE (or Time-Restricted Feeding (TRF) in animals) times food access on the circadian clock [3] to when the body is best prepared to receive food intake. The circadian rhythm represents a 24-h rhythm that plays an important role at the molecular, physiological, and behavioural levels [3,4,5,6]. Its regulation works via clock genes in the suprachiasmatic nucleus, also known as the “central/master” clock which exerts its effects on several secondary clocks distributed throughout the body [7,8,9]. Along with internal regulation, external input such as light and nutrient consumption influences the circadian rhythm resulting in the circadian system integrating both the internal and external state of the body [3]. Notably, macronutrient content and time of food consumption have an influence on peripheral circadian oscillators [1,10,11,12].

The coordination of food intake by the brain over the circadian cycle means there are daily windows which overlap with the active phase of the body when the food intake can be best received. Feeding outside this time may promote energy storage [3] and has been associated with higher levels of triglycerides, LDL, and total cholesterol in observational studies [13,14] which may lead to poor cardiometabolic health. TRE aims to align the body with the circadian rhythm and is different to other fasting strategies such as intermittent fasting, periodic fasting, and caloric restriction as TRE does not require a reduction in calorie intake [15,16]. A subset of TRE which is often excluded from TRE reviews includes Ramadan, a religious fasting during which Muslims restrict dietary intake from dawn to sunset with 2–3 meals taken between sunset and predawn time [17]. This results in a variable fasting period depending on time of year and geographical location during which energy consumption is limited to the inactive phase. This is coupled with changes in sleep/wake patterns which has a potential to disrupt circadian rhythmicity [18,19,20].

The effects of circadian rhythm are seen in several metabolic and hormonal rhythms including insulin sensitivity, plasma lipids, cortisol, insulin, and growth hormone. Moreover, cortisol and melatonin are hormonal signals involved in the communication between the master clock and peripheral oscillators along with other factors such as body temperature and the autonomic nervous system [1]. Cortisol is the hormonal end-product of the hypothalamus–pituitary–adrenal (HPA) axis and plays a major role in an organism’s adaptation and adjustment of homeostasis in response to intrinsic and extrinsic challenges. As a result, cortisol mediates many metabolic processes that include enhancing cardiovascular output/respiration, mobilization of energy and increases in energy delivery rates to the brain and muscles [21]. A 24-h rhythm of episodic release is observed in relation to cortisol under normal sleep-wake conditions, with levels rising in the latter stages of sleep, to a peak in the early morning during the active phase, followed by declining levels during the day, with lowest levels reported shortly after falling asleep [22]. Along with cortisol release in anticipation of the active phase, cortisol levels are also affected by feeding. During day time, food intake has resulted in acute increases in salivary cortisol levels that begin toward the end of the meal (30-min), peak approximately 1 hour after the start of the meal and then return to pre-meal levels approximately 2 h postprandially [23]. This is coupled with food-anticipatory changes in cortisol shown by rodent studies where feeding was restricted to the resting phase resulting in both a rise in cortisol at dusk mediated by the master lock, and a pre-prandial peak [24]. This variation in cortisol has been suggested as “the most robust circadian rhythm of all blood constituents in mammals”. Melatonin similarly plays a role in circadian rhythm, shifting the rhythm to align with environmental cues of light and darkness; it also promotes sleep onset. Both cortisol and melatonin represent markers of circadian rhythm.

Chrono-nutrition is an emerging field of nutritional science that aims to understand how timing of food-intake may impact our health by affecting our circadian rhythm. Therefore, this paper aimed to examine the effects of two patterns of TRE, traditional TRE with a restricted window of eating during the active phase, and Ramadan fasting, during the inactive phase, on two markers of circadian rhythm, cortisol and melatonin.

## 2. Materials and Methods

This systematic review is reported according to the Preferred Reporting Items for Systematic Reviews and Meta-Analyses (PRISMA) Statement [25].

### 2.1. Search Strategy & Inclusion/Exclusion Criteria

A search was performed on PubMed, and Web of Science (all databases) up to December 2020 using search terms relating to “time-restricted eating”, “intermittent fasting”, “cortisol”, “melatonin”, and “orexin”. The full search strategy is available in Supplementary Materials. Randomised controlled trials, and observational studies that met the following criteria were included: study participants over 18 years of age who underwent TRE, outcomes including cortisol and melatonin, and no restrictions were placed on sample size. Systematic reviews and case reports were also excluded. Additionally, any studies not written in English were excluded. Studies were also excluded if participants were on medications that modified our primary outcomes (e.g., hydrocortisone) but no restrictions were placed on the physiological status of the samples. Two independent authors (S.C., S.B.) screened the titles and abstracts of articles against the inclusion and exclusion criteria. Subsequently, full texts were reviewed against eligibility criteria for final selection. If full texts were not available, authors of the respective papers were contacted. Any disagreements between the two authors were resolved by discussion.

### 2.2. Data Extraction

A pre-designed excel sheet was used to extract and organise the data into categories by two independent authors (S.C., S.B.). These included (1) study characteristics i.e., author, title, intervention type, intervention details, follow up period, (2) participant i.e., number of participants, body mass index (BMI), age, (3) outcome details: type of cortisol measured (serum/plasma/urinary/saliva), and cortisol and melatonin response.

### 2.3. Quality Assessment

Quality assessment of all studies was performed using the Newcastle-Ottawa scale for cohort studies [26] (three studies) [27,28,29], the NIH quality assessment tool for before-after (Pre-Post) studies without control group [30] (eight studies) [31,32,33,34,35,36,37,38], and the Cochrane Risk of Bias tool for randomised studies [39] (three studies) [40,41,42].

## 3. Results

### 3.1. Screening

**Figure 1:**
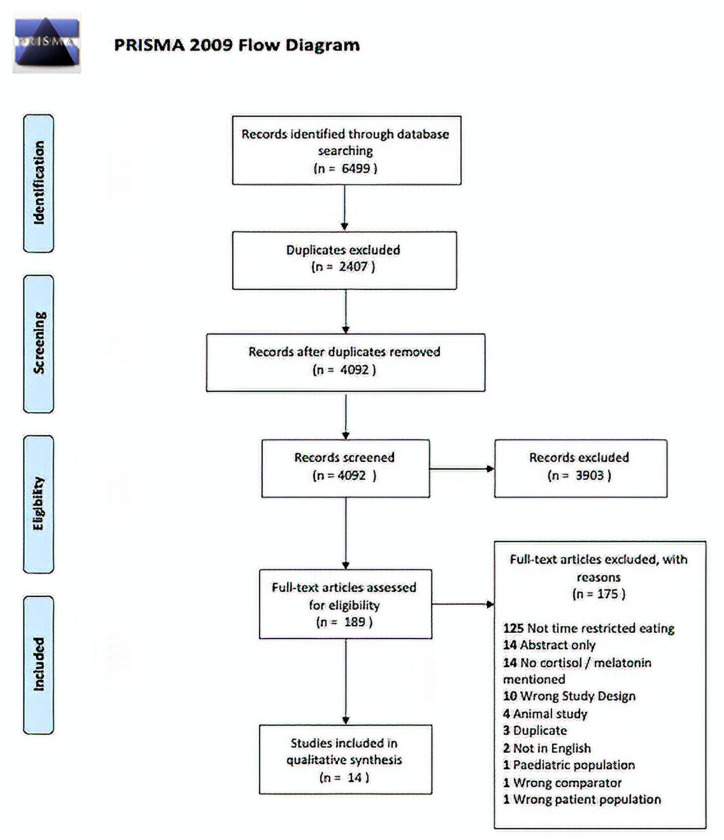
PRISMA 2009 Diagram for included studies.

The search strategy resulted in 4092 studies once duplicates were removed. Of these, 3902 were excluded in title and abstract screening as they did not meet the selection criteria. Thus, 191 articles were retrieved for full text screening. Of these, 14 records met eligibility requirements [27,28,29,31,32,33,34,35,36,37,38,40,41,42] (Figure 1). The characteristics of these studies are summarized in Table 1 and Table 2. Only one paper describing orexin [43] met the inclusion criteria which led to author consensus to limit the data synthesis for cortisol and melatonin outcomes.

### 3.2. Quality Assessment

Using the NHI tool, the primary category that could lead to bias was the lack of a sufficient sample size, as a majority of the studies (7/8) did not assess the power of the study. Moreover, only 4/8 studies used a time-series design to ensure the outcome was accurately measured. In the Newcastle Ottawa Scale, there were issues in “ascertainment of exposure” as 2/3 studies used self-reporting from participants to ascertain the intervention. The Risk of Bias tool revealed a “high” Risk of Bias for the per protocol study and “some concerns” for the two that followed an intention-to-treat analysis. Full results can be found in Supplementary Materials (Table S1).

### 3.3. Summary of Studies

Characteristics of included studies are described in Table 1. Of the 14 studies identified in our literature review, 13 reported results on cortisol, and four on melatonin (Table 1). We grouped the traditional TRE and Ramadan studies separately as the interventions take place during different parts of the circadian rhythm (TRE during the active phase and Ramadan in the inactive). Ten of the studies used TRE in the context of Ramadan whilst four followed non-Ramadan TRE protocols.

#### 3.3.1. Cortisol Changes after Time-Restricted Feeding (Ramadan)

Two of the three [33,34,37] studies examining the changes in the circadian rhythm of cortisol during Ramadan observed an abolishing of the circadian pattern of cortisol. Bahijri et al. [33] remarked lower levels of morning cortisol during Ramadan that were borderline non-significant compared to the non-fasting month (*p* = 0.06). Both Bahjiri et al. and Haouari et al. [33,37] remarked significantly higher evening/night levels of cortisol (*p* < 0.01) and a decrease in the variability of cortisol over 24 h due to lower morning levels as well as higher night levels of cortisol (Table 1). On the other hand, Bogdan et al. [34] remarked that cortisol maintained its circadian rhythmicity but with a biphasic pattern. It had an evident rise in serum concentration starting at 12:00 h and a plateau between 16:00 h and 20:00 h, i.e., at the time of the first meal following the daytime fasting period. Neither Bogdan et al. nor Haouari et al. found any significant changes in 24-h serum cortisol during Ramadan compared with the control.

Al-Rawi et al., Chennahoui et al., Brini et al., and Vasaghi-Gharamaleki et al. [31,35,36,38], only measured late morning cortisol (Table 1). Al-Rawi et al. and Chennahoui et al. found no significant changes in cortisol level [31,36]. Conversely, Vasaghi-Gharamaleki’s study found that morning cortisol concentration and output significantly decreased compared to baseline during Ramadan and this decrease lasted 3 weeks after Ramadan (*p* < 0.05) [38]. Brini et al. found a similar decrease in morning cortisol at the end of Ramadan in comparison to pre-Ramadan levels (*p* < 0.005) [35]. Moreover, Zangeneh et al. [28] observed a significant decrease in the fasting group of the women with PCOS and Dikensoy et al. [27] observed a significant rise in cortisol in the fasting group of pregnant women. However, neither of these two studies reported the time of sample collection.

#### 3.3.2. Melatonin Changes after Time-Restricted Feeding (Ramadan)

Of the studies examining TRF during Ramadan, 4/10 examined the changes in melatonin [31,32,34,36]. All four studies found a statistically significant reduction of melatonin during Ramadan (Table 1). Almenessier et al. [32] found a statistically significant decrease during Ramadan compared to baseline but not during fasting outside Ramadan (FOR). Two out of the four studies examined the effect of Ramadan on the circadian pattern, and both found that the same circadian pattern was maintained before and during Ramadan despite an overall reduction [32,34].

#### 3.3.3. Cortisol Changes after Time-Restricted Feeding (Non-Ramadan)

All studies reviewed recorded cortisol shifts in response to time restricted eating, albeit over different time frames and different cortisol recording methods. Two studies limited their TRE protocol to skipping breakfast and the other two allowed for more flexible protocol where morning eating was either accepted within the parameters of the study or morning eating was part of the protocol itself, and evening eating was prohibited. Not all the recorded cortisol shifts reached statistical significance, (Table 2) and not all were T-tested. Some of the studies were accompanied by additional interventions stacked with TRE, such as caloric restriction, resistance training, and additional stress factors. Results regarding cortisol changes were mixed and often contradictory. None of the non-Ramadan TRE studies examined effects on melatonin levels.

## 4. Discussion

Our review identified an interesting relationship with cortisol in response to various TRE practices. Notably, it appeared that Ramadan practice of TRE resulted in a reduced waking response of cortisol, and in most cases a statistically significant increase relative to non-Ramadan in the evening. The Ramadan studies also found significant reductions in levels of nocturnal melatonin during Ramadan compared to baseline. In the non-Ramadan TRE studies, it was found that skipping dinner led to a decreased evening cortisol level, and, potentially, an elevated morning level, though in not all cases was waking time accounted for. Meanwhile, skipping breakfast led to a relative increase in midday cortisol. This relationship appears to hold true regardless of whether the TRE protocol also engenders caloric restriction.

### 4.1. Circadian Rhythm of Cortisol

The results for the non-Ramadan TRE studies indicate that depending on the time of day one omits from the meal schedule, there is a shift in the diurnal curve of cortisol secretion to the right. It is well precedented that fasting to the point of glycogen depletion leads to dependence on gluconeogenesis to produce energy, the two hormones that control this mechanism being cortisol and growth hormone. This could therefore explain the cortisol shifts we observed in our review, though whether the participants spent enough time unfed to the point of glycogen depletion is not clear. What also was not always clear was whether cortisol was measured before or after a meal. This is relevant, as cortisol levels can shift dramatically in response to a meal, and the lack of uniformity in study design may have obfuscated our results in this regard. For example, rather paradoxically, increased cortisol levels can also occur by taking, rather than missing, a meal [44]. The mechanisms behind this are not well understood, but hypotheses surround the phenomenon point to: the complex endocrine interplay with insulin response and glucocorticoid secretion [45], and amino acids such as tryptophan, which is affected by dietary intake, have also been implicated in increased cortisol secretion [46]. Indeed, this phenomenon was observed in a study we reviewed by Witbracht et al. (2015) [29], who observed that females who skipped breakfast exhibited significantly higher cortisol postprandially—especially at midday—than did the controls. This suggests that the delayed action by the HPA-axis to a skipped meal produces a compensatory effect on the next meal. This could be the result of stacked effect, whereby rise in cortisol postprandially stacks on elevated cortisol that was already present because of the HPA-axis sympathetic response and or potential gluconeogenesis. Therefore, a sustained rise in cortisol to midday and/or postprandially could represent a survival response to utilise endogenous energy and then stimulate further food intake [29].

Jamshed et al. [40] reported significantly lower evening cortisol in those who skip dinner as their TRE strategy. This is interesting, because in this study blood results were always taken pre-prandially, and therefore the differences cannot be the result of postprandial cortisol augmentation in the control group. A possible explanation for this is the cortisol anticipatory response, in which an elevation in cortisol levels are reported in anticipation of a meal [47]. As the intervention group was skipping the evening meal as their TRE strategy, they were unlikely to have experienced the related pre-meal increase of cortisol, that may have been part of the difference between cortisol levels in the groups. Similar anticipatory responses are reported with cortisol to stress responses, with athletes anticipating sporting competition reporting significant anticipatory cortisol responses [48]. Dinner-skipping resulted in significantly reduced evening cortisol and non-significantly raised morning cortisol which suggested that TRE increased the amplitude of the cortisol rhythm. Conversely, breakfast skipping resulted in a blunted/flat diurnal cortisol pattern with significantly reduced morning cortisol. This blunting indicates a dysfunctional HPA axis and may be associated with poor cardiometabolic outcomes [29,49,50].

These findings are critical for the practical implementation of TRE. We have seen the benefits of restricting the feeding window for longevity and metabolic health in both human and animal studies [1,51,52,53,54,55]; however, the question then arises of whether there is an optimal feeding window that allows for the greatest benefits of TRE [56].

We hypothesised that whether one chooses early or late TRE can determine the optimal time of alertness and sleep. Early TRE, which involves skipping dinner, may allow for greater alertness in the morning as the lower levels of cortisol at night can improve sleep quality and higher morning cortisol raises alertness [57], optimising wakefulness and productivity. Conversely, for greater alertness mid-day, it may be beneficial to skip breakfast for an intensified cortisol response mid-day as the cortisol curve shifts to the right. However, the early TRE study in question involved an eating window of 8 a.m.–2 p.m. A previous study examining overnight fasting found that for those undertaking early overnight fasting, a longer fasting duration was associated with lower odds of overweight and obesity; but among those with late fasting, longer fasting was associated with higher odds of overweight and obesity [58]. This further supports the need for more research to investigate the difference between early and late time eating windows that are designed to be more socially acceptable in order to confirm and translate the proposed benefits of TRE into practice.

Stratton et al. [42] compared TRE to a normal dietary pattern, both of which had a 25% caloric deficit. Higher concentrations of cortisol were found following the normal diet compared to TRE diet (*p* = 0.018). The authors speculated that this may have been because fasting alters the normal circadian rhythm of cortisol rises and falls and that the TRE could have altered the normal spike seen at the time of day when cortisol was assessed. Similarly, a recent abstract examining the effect of an 8-week TRE intervention on firefighters found significantly reduced levels of salivary cortisol in response to a simulated firegrounds test (FGT) which may have implications for a reduced stress response [59]. Moreover, McAllister at al [41] compared an ad libitum (eating to satiation) TRE diet to an isocaloric TRE diet (defined as +/− 300 kcal difference from baseline) finding no significant impact from the TRE interventions. However, caution must be taken when interpreting findings of these papers as cortisol levels are highly dependent on the time of day that they are taken; McAllister et al. took blood samples between 5 and 9 a.m. following at least an 8 h fast, and Stratton et al. did not specify the exact time at which blood was taken, apart from being within +/− 2 h of the original time.

The Ramadan-based TRE studies suggest that the religious fast may lead to a disruption of the circadian rhythm with lower morning cortisol levels and higher evening cortisol levels. However, it remains unclear whether this is indeed a disruption or delay in the circadian rhythmicity of cortisol. The review by BaHammam et al. [60] noted that the shift delay in the biological clock seen in some Ramadan studies is multifactorial and impacted by sleep/wake schedule, sleep duration, light exposure, caloric consumption, and energy expenditure [60]. They concluded that restricting mealtime to the early evening and predawn times, accompanied by good sleep during Ramadan, would not affect the biological clock.

A flattening of the circadian cortisol rhythm is associated with conditions such as cancer, cardiovascular disease, obesity, and other metabolic disorders [3,33,61,62]. However, there is insufficient evidence as to whether the changes in circadian rhythmicity of cortisol during Ramadan lead to these consequences [63,64,65,66]. For example, a recent systematic review and meta-analysis found that Ramadan TRF is effective in decreasing body weight (−0.353; 95% CI [−0.651, −0.054], *p* = 0.02) and relative fat mass (−0.533; 95% CI [−1.025, −0.04], *p* = 0.034) [67], and another meta-analysis found positive effects on cardiometabolic risk factors such as triglycerides, total cholesterol, LDL, VLDL, HR, DBP, and increases HDL [68]. Given the unclear link between the disruption of cortisol rhythmicity and cardiometabolic outcomes in Ramadan studies, further work should be done to understand whether there is any direct impact related to the circadian rhythm. Peak levels of glucocorticoids are synchronised with the onset of the active phase which aids arousal. Higher levels of cortisol at night could thus be associated with reduced sleep quality. A previous Ramadan study controlling for lifestyle factors such as, but not limited to, the sleep/wake schedule found that fasting during and outside Ramadan led to reduced REM sleep when compared to baselines, concordant with other studies that did not control for lifestyle factors [69,70,71]. A proposed mechanism for this was the nocturnal rise in cortisol and insulin. Another systematic review and meta-analysis confirmed that Ramadan led to an approximately 1 h reduction of sleep and a 1 point increase in the ESS score [72]. Some studies have associated sleep deprivation with an increase in energy intake [73,74,75] and obesity, meaning that TRE during the non-active phase could result in over-eating.

Previous reports have stated that single-point cortisol values can be misleading in many Muslim countries during or shortly after Ramadan. Hence, it is better to judge cortisol response to Ramadan on the overall secretion distribution. Overall, it is well precented and observed in this study that diurnal IF through Ramadan causes very apparent circadian shifts in cortisol secretion. This is also very dependent on the time of year Ramadan falls and what country the study takes place in. For example, Ramadan, when practised in a non-equatorial country during the winter, will have a far shorter diurnal fasting duration, and also a probably non-significant change in bed-time than a summer Ramadan; because of this, geography and time of year will always confound circadian studies predicated on Ramadan diurnal IF. Instances in which we observed more pronounced circadian related shifts in cortisol in participants may possibly have been during summertime, though this did not appear to be a recorded factor. More generally, only consuming one’s meals during the night-time is a phenomenon very much observed in nocturnal species, and it is hypothesised that circadian gene expression can be mediated by the time of consumption of meals. Animal models have indicated that eating during the wrong time of the day (for one’s species) can cause a misalignment between the peripheral circadian clocks and the central super-chriamastic nucleus clock. For example, Damiola et al. [76] showed that temporal feeding restriction under light–dark or dark–dark conditions can change the phase of circadian gene expression in peripheral cell types by up to 12 h. Indeed, similar shifts in cortisol secretion have been observed in non-Muslim night shift workers [77].

### 4.2. Melatonin

All Ramadan studies examining melatonin found a post-fasting reduction in melatonin. Bogdan et al. [34] remarked that melatonin maintained its circadian rhythmicity but with decreased amplitude and a trend towards a phase delay. They hypothesised that the flatter slope of the melatonin increase may be due to the prolonged exposure to artificial light during Ramadan [34]. However, other proposed mechanisms include the nocturnal increase in cortisol levels during Ramadan [69].

There is a paucity of studies examining the effects of different dietary patterns on melatonin. However, the central hypothesis of effects of melatonin on insulin is that elevated melatonin during glycaemic challenges such as taking a meal inhibits insulin release and impairs glucose tolerance [78] with its effects mediated through the melatonin receptors (MT1 and MT2) [79]. These results remain to be confirmed in larger clinical trials; however, the practical consequence of these findings could be the reduction of insulin production in response to meals taken late at night, close to bedtime. This effect is further accentuated in humans carrying a mutant allele for melatonin receptor 1B (MTNR1B), expressed in insulin-producing pancreatic islets. Carriers of this mutation have increased risks for hyperglycaemia and type 2 diabetes [80,81,82]. It has therefore been suggested to be advantageous to avoid meals for the 2–3 h before going to bed and for an hour after waking up during which melatonin levels are elevated [53]. This further supports indications for TRE which would restrict eating in that time period.

This could also carry significant implications for people undertaking Ramadan fasting as increased melatonin is produced during the circadian night, suppressing insulin response when meals are taken resulting in a dysregulated glycaemic response. The reduction in melatonin seen in the Ramadan studies could represent a compensation for this mechanism, but more studies examining the effects of TRE on melatonin levels are required to confirm this hypothesis.

### 4.3. Strengths and Limitations

This study has certain limitations to address. In terms of the methodology, we could have expanded our search to other databases such as Cochrane and Google Scholar. For our analysis, we were unable to pool the data on cortisol and melatonin due to the heterogeneity in study design, data collection methods, and parameter type collected. Moreover, studies assessed levels of cortisol and melatonin at different times of day and did not report what time of year Ramadan fasting was undertaken. Further limitations include the inclusion of the studies by Dikensoy et al. [27] and Zangeneh et al. [28] which included women with PCOS and pregnant women, respectively, and the specific pathological and physiological implications of the included patients could introduce heterogeneity in the findings. Therefore, further exclusion criteria on pregnancy, pathology, and athletic status should have been considered. There is a limited amount of literature examining the effect of TRE on circadian markers which proved analysis to be difficult. Other limitations include the quality of the included studies, most of which had small sample sizes and did not use a time-series design. Some Ramadan studies also did not control for confounding factors that can affect serum parameters, such as sleep duration and timing, total energy expenditure, and light exposure [27,28,31,33,35,36,37,38]. Caution must also be taken in terms of generalisability due to the small sample size of the studies. However, this study has several strengths. We adhered to a strict systematic review protocol and screened 4092 papers to ensure we included all relevant papers. We also included both TRE and Ramadan papers to provide a comprehensive understanding of how limiting eating windows affects biomarkers of the circadian rhythm in both the active and inactive phase. We have also included an extensive analysis of the physiological methods that could contribute to our findings. Future studies can broaden their scope to consider growth hormone which along with cortisol impacts the circadian rhythm [83], and previous studies have examined the impact of Ramadan fasting on growth hormone [84,85]. Moreover, there is potential for further comparative studies to be conducted on the impact of a breakfast-skipping TRE protocol in comparison to dinner-skipping, as well as comparing TRE during the active phase compared to the inactive phase of the circadian rhythm.

## 5. Conclusions

This paper found a potential blunting of the circadian cortisol rhythm during Ramadan and a reduction in melatonin which could lead to poor sleep duration and quality, as suggested by these markers of circadian rhythm. There was a paucity of research examining the effects of TRE intervention during the active phase on cortisol and melatonin. However, the contrasting effect of dinner and breakfast-skipping should be further examined in a controlled study to ascertain whether timing the feeding window indeed has an impact on circadian rhythmicity. In order to better understand the effects of meal timing on health outcomes, further studies examining TRE during the inactive and active phases of the circadian rhythm should be conducted under controlled conditions.

## Figures and Tables

**Table 1:** Study Characteristics of Included Papers on Ramadan.

Citation	Study Conditions	N	Population Characteristics (Gender, Age, Body Mass Index (BMI))	Cortisol Type	Cortisol Response	Melatonin Response
Al Rawi et al. 2020 * [31]	Each subject’s values 1 week before Ramadan (T1) were compared to outcomes at the end of the fourth week of Ramadan (after completing 28 consecutive days of fasting, or T2). During Ramadan, study subjects abstained from all food and drink (including water) from dawn to sunset. The daily fasting duration during this study was approximately 15 h. Subjects were assessed in the late morning (11 a.m.–1 p.m.)	57	17 females, 40 males Average age (years): 38.4 ± 11.2 years Average BMI: 29.9 kg/m^2^	Salivary	At the end of Ramadan, cortisol did not change compared to the levels assessed in the pre-fasting state (*p*-value “non-significant”).	At the end of the fasting month, serum melatonin was significantly decreased (*p* < 0.001) compared to baseline by 39%.
Almeneessier et al. 2017 [32]	Participants reported to the Sleep Disorders Center (the laboratory) on four occasions: (1) adaptation, (2) 4 weeks before Ramadan while performing Islamic intermittent fasting for 1 week (fasting outside Ramadan (FOR)), (3) 1 week before Ramadan (non-fasting baseline (BL)), and (4) during the 2nd week of Ramadan while fasting (Ramadan).	8	8 males Average age (years): 26.6 ± 4.9 Average BMI: 23.7 3.5 kg/m^2^.	NA	NA	The melatonin levels followed the same circadian pattern during the three monitoring periods (BL, FOR, and Ramadan). Lower melatonin levels at 22:00 h were found during fasting compared to BL, with a significantly lower level for Ramadan versus BL (*p* < 0.05). No significant changes in the acro-phase of melatonin levels.
Bahijri et al. 2013 [33]	Two intervention periods: Young, Ramadan practitioners were evaluated before and two weeks into the Ramadan. Blood samples were collected at 9.00 a.m. and 9.00 p.m. for measurements of metabolic parameters and cortisol. Saliva was collected every 4 h for 24 h, except when asleep, during both days of blood sampling.	24	5 females, 19 males (one male dropped out) Average age (years): 23.1 ± 1.2 Average BMI (kg/m^2^): 24.6 ± 1.1	Salivary & Serum	Serum: Cortisol circadian rhythm was abolished during Ramadan (*p* = 0.068). Morning level was lower in Ramadan compared with the non-fasting month, but not significantly so (*p* = 0.06). Evening cortisol in Ramadan was significantly higher than during the non- fasting month (*p* = 0.008). This was reflected in a significantly lower a.m./p.m. cortisol ratio during Ramadan (*p* = 0.004) (Table 2). Salivary **: Ramadan resulted in obvious flattening of circadian cortisol secretion during the fasting month compared to the non- fasting month; with lower levels during mid-morning and higher levels in the evening and early morning. Had significantly decreased values in the evening during the non-fasting month (*p* = 0.018), but no such a decrease during the fasting month of Ramadan (*p* = 0.254).	NA
Bogdan et al. 2001 [34]	Two intervention periods: The volunteers were studied twice over a 24-h span: one week before Ramadan (control: end of December) and on the twenty-third day of Ramadan (Ramadan: end of January).	10	10 males Average age (years): 34 ± 3.7 Average BMI (kg/m^2^): NR	Serum	Serum cortisol levels rose in the afternoon, while the morning rise was apparently delayed. A higher morning peak and a sharper decline were observed during Ramadan. On the Ramadan test day, the cortisol rhythm was overtly biphasic, with an evident rise in the serum concentration starting at 12:00 h and a plateau between 16:00 h and 20:00 h, i.e., at the time of the first meal following the daytime fasting period. The morning cortisol peak was higher and steeper during Ramadan than on the control day. No significant increase in 24-h mean concentration of serum cortisol during Ramadan.	The nocturnal peak of melatonin was diminished (*p* < 0.008) and may have been delayed. The melatonin pattern remained circadian during Ramadan.
Brini et al. 2019 [35]	16 basketball players were randomly assigned to one of two training groups: a small-sided game group (GSSG; N. = 8) and a repeated sprint ability group (GRSA; N. = 8. The groups completed a 4-week training program during Ramadan (R, experimental month) and one month after Ramadan (AR, control month), interrupted by 15 days of total recovery, with a frequency of two sessions per week. Data was measured on six occasions: before R (P1), at the end of the second week of R (P2), at the end of R (P3), before the AR training period (P4), at the end of the second week AR (P5) and at the end of the AR training period (P6). Players reported to the laboratory at 09:30 h. Blood samples were collected from the seated athletes at least ~9 h after their last meal and at least 24 h after their last training session.	16	16 males Average age (years): 23.4 ± 3.7 Average BMI (kg/m^2^): 22.6 ± 1.95	Plasma cortisol	G_SSG +_ G_RSA_: Delta variation of cortisol showed a significant decrease in P3 compared to P1 in all subjects (*p* < 0.005) G_SSG_: Cortisol lower in P3 than it was in P1 (*p* = 0.036). G_RSA_: Cortisol was lower in P3 (75.1 +/− 51.3) compared to P1 (136 +/− 136 +/− 48.6) but this change was not significant. AR cortisol (1 month after Ramadan) was higher than cortisol during R after 2 and 4 weeks of training (DP2-P1 vs. DP5-P4: *p* = 0.0008 and DP3-P1 vs. DP6-P4: *p* < 0.0003).	NA
Chennaoui et al. 2009 [36]	In 8 middle-distance athletes a maximal aerobic velocity (MAV) test was performed 5 days before RF (day–5), and on days 7 and 21 of RF. The subjects observed RF and abstained from food and liquids from approximately 05:00 to 19:00 h for 30 days. The length of each fasting day was approximately 13 h. Samples collected 10 a.m.–11 a.m.	8	Participant sex unclear Average age (years): 25.0 ± 1.3 Average BMI (kg/m^2^): NR	Salivary	Cortisol concentration increased only at day 7 (of RF). At day 7 and day 21 of RF, compared with day 5, and before the MAV test cortisol concentration was not statistically different (*p* = NS).	At the end of RF (P2), compared with before RF (P1), serum melatonin decreased (*p* < 0.05)
Dikensoy et al. 2009 [27]	Thirty-six consecutive healthy women with uncomplicated pregnancies of 20 weeks or more, who were fasting during Ramadan, were included in the study group (group 1). The control group (group 2) consisted of 29 healthy pregnant women, who were not fasting during the study period. Maternal blood samples were obtained 1 week prior to Ramadan and on 20th days of fasting	65	Women (pregnant women, 36 fasting during Ramadan, 29 not fasting) Average age (years): NRAverage BMI (kg/m^2^): NR	Serum	In the fasting group, the maternal serum cortisol levels on day 20 were significantly higher than the initial levels obtained 1 week prior to Ramadan (*p* < 0.05).	NA
Haouari et al. 2008 [37]	Three intervention periods: Participants were examined on days 7 (D7) and 21 (D21) of Ramadan and one week before Ramadan as control (D0). The average duration of the fast was about 12 h Five millilitres of venous blood were drawn at 9:00, 13:00, 17:00, 21:00, 01:00, and 05:00	36	36 males Average age (years): 24 ± 1.6 Average BMI (kg/m^2^): average NR; 18.5 < BMI ≤ 24.9	Serum	The analysis of cortisol circadian showed a significant sinusoidal wave form of the three curves, both for the days of fasting (D7 and D21) and for the control day (D0) (*p* < 0.001), with a morning peak. Data showed no significant changes in the 24-h mean or in the amplitude and time of peak compared with the control day. However, results show that nocturnal cortisol levels during Ramadan were higher than during the control period (*p* < 0.01) at midnight on both D7 and D21. Curve of D7 showed a very slight advance, by one hour and 18 min accompanied by a remarkable decrease (*p* < 0.001) in the variability during the 24 h.	NA
Vasaghi-Gharamaleki et al., 2014 [38]	Cross-sectional study Saliva was collected 2 weeks before the beginning of Ramadan (BR), during the first week (R1), middle (R2), the last week (R3) of Ramadan and 3 weeks after Ramadan (AR). Collection during 8 a.m.–9 a.m., no measure of evening cortisol.	30	30 males Average age (years): NR; Age range: 30–76 years Average BMI (kg/m^2^): NR	Salivary	Morning cortisol concentration and output significantly decreased compared to baseline in R1, R2, R3 and AR. Decrease in saliva cortisol level lasted 3 weeks after Ramadan (*p* < 0.05)	
Zangeneh et al. 2015 [28]	A total of 40 women who were aged 20–40 years and known cases of PCOS and had no other medical diseases were included in the study. They were divided into two groups as follows: (i) study group (*n* = 20) who participated in Ramadan fasting and (ii) control group (*n* = 20) who did not participate in fasting. Variables were evaluated before and after Ramadan	40	40 females with PCOS Average age (years): Ramadan fasting: 29.40 ± 4.60 Non fasting: 28.80 ± 3.86 Average BMI (kg/m^2^): average NR; 18.5 < BMI ≤ 24.9	Serum	Cortisol hormone concentration decreased in the fasting group (*p* = 0.049).	NA

* Females exempt from fasting during menstruation; **: samples collected at different times of day; NR: not reported. PCOS: polycystic ovarian syndrome. DIF: diurnal intermittent fasting.

**Table 2:** Study Characteristics of Included Papers on Day-time Time Restricted Eating.

Citation	Study Conditions	N	Population Characteristics	Cortisol Type	Cortisol Response
Jamshed et al. 2019 [40]	Eleven overweight adults participated in a 4-day randomized crossover study where they ate between 8 a.m. and 2 p.m. (early TRF (eTRF)) and between 8 a.m. and 8 p.m. (control schedule). Blood samples were collected in the fasting state at 20:00 on day 3 (evening, p.m.) and immediately after exiting the chamber at ~07:30 on day 5 (morning, a.m.). The evening blood draws were taken immediately before dinner in the control arm.	11	4 females, 7 males Average age (years): 32 ± 7 years. Average BMI (kg/m^2^): 30.1 ± 2.7		Increased morning cortisol levels but was not significant (*p* = 0.10), significantly decreased evening cortisol levels (*p* = 0.03).
McAllister et al. 2020 [41]	8 h feeding window. No restrictions on what time of day this was to be performed. Pre and Post TRE results were recorded, groups separated into isocaloric and no caloric restriction. Blood samples were collected between 05:00 and 09:00 following at least an 8 hr fast via venipuncture and finger prick. 28-day protocol.	22	22 adult males. Average age (years): 22 ± 2.5 Average BMI (kg/m^2^): 28.5 ± 8.3.		TRE + No Caloric Restriction led to reduction in Cortisol (39.8 to 37.3 ug/dL, not T-tested). Isocaloric TRE led to rise in cortisol (31.8 to 35.2 ug/dL, not T-tested).
Stratton et al. 2020 [42]	Results for Pre and Post TRE (breakfast skipping) + 25% Caloric restriction + regular resistance training vs. No time restriction +25% caloric restriction + resistance training. Cortisol assessments were taken at the same approximate time pre- and post-intervention (±2 h), time from the waking hour was not quantified, which may have also affected the measurement	26	26 Males Average age (years): 22.9 ± 3.6 Average BMI (kg/m^2^): not provided		TRE + Caloric restriction + reg. resistance training led to reduction in cortisol (118.3 to 106.1 ng/mL). No time restriction led to increase in cortisol (119.2 to 150.7 ng/ML). Both *p* = <0.05
Witbracht et al. 2015 [29]	Observational Study acquired those already performing TRE (breakfast skippers). Salivary cortisol taken 6 times throughout the day + waking and bedtime for one day for both case and control.	65	65 Females Age range: 18–45 (mean not provided) Average BMI (kg/m^2^): 24.8 ± 6.7		TRE (breakfast skippers) demonstrated decreased morning (waking) cortisol, elevated midday mid-day cortisol, and no significant evening differences compared to the control group.

## Supplementary Materials

The following are available online at https://www.mdpi.com/article/10.3390/nu13082525/s1, File 1: Search Terms, Table S1: Quality Assessment of Included Studies.
