# Supplementary material for: The Window Matters: A Systematic Review of Time Restricted Eating Strategies in Relation to Cortisol and Melatonin Secretion
Source: Nutrients. 2021 Jul 23;13(8):2525. doi: 10.3390/nu13082525 (PMC8399962; doi:10.3390/nu13082525)
Supplement: Supplementary file 1 [file nutrients-13-02525-s001.zip › nutrients-1259843-supplementary/Supplementary Materials.pdf]

Supplementary Materials

Pubmed Search Terms: 2654

((((((((((fasting*[MeSH Terms]) OR (time restricted eating*[MeSH Terms])) OR (time restricted feeding*[MeSH Terms])) OR (intermittent fasting*[MeSH Terms])) OR (caloric restriction*[MeSH Terms])) OR (fasting*[Text Word])) OR (time restricted eating*[Text Word])) OR (time restricted feeding*[Text Word])) OR (intermittent fasting*[Text Word])) OR (caloric restriction*[Text Word]))) AND

(((((((((((hydrocortisone*[MeSH Terms]) OR (melatonin*[MeSH Terms])) OR (orexin*[MeSH Terms])) OR (hydrocortisone*[Text Word])) OR (cortisol*[Text Word])) OR (salivary cortisol*[Text Word])) OR (urinary cortisol*[Text Word])) OR (serum cortisol*[Text Word])) OR (melatonin*[Text Word])) OR (orexin*[Text Word]))) OR (hypocretin*[Text Word]))

Web of Science all databases: 3845

Search History:

Set	Results	Save History / Create Alert Open Saved History	Combine Sets<br>AND OR<br>Combine	Delete Sets<br>Select All<br>Delete
# 3	3,845	#2 AND #1<br>*Databases= WOS, KJD, MEDLINE, RSCI, SCIELO Timespan=All years*<br>*Search language=Auto*	☐	☐
# 2	198,853	TS =(hydrocortisone* OR cortisol* OR serum cortisol* OR urinary cortisol* OR salivary cortisol* OR melatonin* OR orexin* OR hypocretin*)<br>*Databases= WOS, KJD, MEDLINE, RSCI, SCIELO Timespan=All years*<br>*Search language=Auto*	☐	☐
# 1	194,824	TS = (fasting* OR time restricted feeding* OR time restricted eating* OR intermittent fasting* OR caloric restriction*)<br>*Databases= WOS, KJD, MEDLINE, RSCI, SCIELO Timespan=All years*<br>*Search language=Auto*	☐	☐
			AND OR<br>Combine	Select All<br>Delete
